# Progress, Key Issues, and Future Prospects for Li‐Ion Battery Recycling

**DOI:** 10.1002/gch2.202200067

**Published:** 2022-06-22

**Authors:** Xiaoxue Wu, Jun Ma, Junxiong Wang, Xuan Zhang, Guangmin Zhou, Zheng Liang

**Affiliations:** ^1^ Frontiers Science Center for Transformative Molecules School of Chemistry and Chemical Engineering Shanghai Jiao Tong University Shanghai 200240 China; ^2^ Shenzhen Geim Graphene Center Tsinghua‐Berkeley Shenzhen Institute & Tsinghua Shenzhen International Graduate School Tsinghua University Shenzhen 518055 China

**Keywords:** battery recycling, direct regeneration, hydrometallurgical processes, lithium‐ion batteries, pyrometallurgical processes

## Abstract

The overuse and exploitation of fossil fuels has triggered the energy crisis and caused tremendous issues for the society. Lithium‐ion batteries (LIBs), as one of the most important renewable energy storage technologies, have experienced booming progress, especially with the drastic growth of electric vehicles. To avoid massive mineral mining and the opening of new mines, battery recycling to extract valuable species from spent LIBs is essential for the development of renewable energy. Therefore, LIBs recycling needs to be widely promoted/applied and the advanced recycling technology with low energy consumption, low emission, and green reagents needs to be highlighted. In this review, the necessity for battery recycling is first discussed from several different aspects. Second, the various LIBs recycling technologies that are currently used, such as pyrometallurgical and hydrometallurgical methods, are summarized and evaluated. Then, based on the challenges of the above recycling methods, the authors look further forward to some of the cutting‐edge recycling technologies, such as direct repair and regeneration. In addition, the authors also discuss the prospects of selected recycling strategies for next‐generation LIBs such as solid‐state Li‐metal batteries. Finally, overall conclusions and future perspectives for the sustainability of energy storage devices are presented in the last chapter.

## Graphical abstract

The necessity for battery recycling, various Li‐ion battery recycling technologies including pyrometallurgical, hydrometallurgical, direct repair, and regeneration methods, and recycling strategies of solid‐state Li‐metal batteries are summarized and discussed.

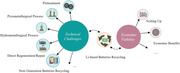

## 1 Introduction

Since 1990s, lithium‐ion batteries (LIBs), as the representative technology for renewable energy storage, have dominated the current market due to their high energy density, high power density, and long life‐span.^[^ ^1^, ^2^ ^]^ For example, LIBs have been used extensively in portable electronics, electric vehicles, and large‐scale grids storage, which help greatly mitigate the use of fossil fuel and the emission of CO_2_.^[^ ^3^, ^4^, ^5^ ^]^ Nevertheless, there exists a key issue—the energy stored inside LIBs is renewable, whereas the raw materials from massive mineral mining to fabricate LIBs are not renewable at all. Estimations expect that the LIBs global market is undergoing an enormous growth from 259 to 2500 GWh within the years 2020–2030 by an average of 25.4% per year.^[^ ^6^, ^7^ ^]^ This indicates a drastically rising demand for raw materials of LIBs, and a drastically rising number of spent LIBs generated due to limited‐service life.^[^ ^8^, ^9^, ^10^ ^]^ Based on this analysis, if a major portion of raw materials to fabricate LIBs could be extracted from spent LIBs instead of from mineral mining, numerous issues including those from economic, environmental, sustainable, and geographical aspects could all be tackled at the same time. It would make LIBs the real crucial factor to unlock renewable energy since the entire process of LIBs including fabrication and employment are all sustainable.^[^ ^11^ ^]^ Fortunately, there are enough spent LIBs generated each year for us to recycle and extract raw materials/precursors from them. According to a report, by the year 2030, global spent LIBs will reach 11 million tons, where the recycling market will extend to $23.72 billion.^[^ ^12^ ^]^

Therefore, LIBs recycling is becoming urgent and vital. Several agencies and institutions over the world have already focused on this topic and initiated some efforts. For example, policies for spent LIBs recycling have been established in some countries like the US, Germany, Japan, and China.^[^ ^6^ ^]^ In addition, ReCell Center led by Argonne National Laboratory has set core principles for sustainable recycling, including the design of novel recyclability, direct recycling/repair/regeneration, and recovery of other high‐value‐added components.^[^ ^13^ ^]^

In this review, we systematically summarize and assess LIBs recycling from the perspectives of necessity (such as economy, environment, sustainability, and geography), current (such as pyrometallurgical and hydrometallurgical methods), and novel (such as direct regeneration/repair methods) recycling technologies. We also discuss the viability of implementing the current recycling technologies in next‐generation Li‐based batteries. Finally, we present challenges and future prospects of LIBs recycling technologies.

## 2 Necessity of LIBs Recycling

### 2.1 High‐Value Resources in Spent LIBs

**Figure 1:**
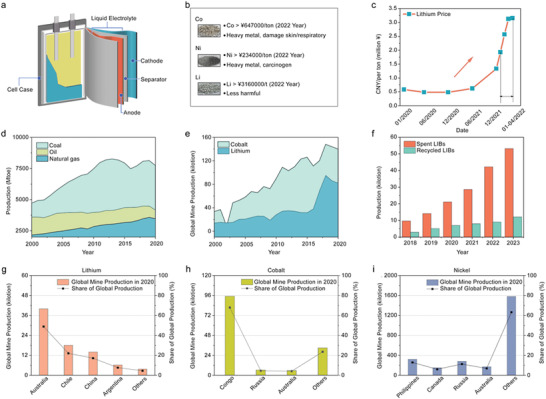
a) Schematic showing each component of LIBs. b) Market value of major metal species in LIBs: cobalt, nickel, and lithium. c) Lithium price change from 2020 to 2022. d) Global fossil fuel (coal, oil, natural gas) and e) mineral mining (cobalt, lithium) production from 2000 to 2020. f) China LIBs recycling industry market analysis from 2018 to 2023. Global distribution and production of main LIBs raw materials: g) lithium, h) cobalt, and i) nickel in 2020. Metal prices are the latest prices obtained from the Shanghai Nonferrous Metals Network. Global fossil fuel production data is obtained from BP Statistical Review of World Energy 1965–2020. China LIBs recycling data is obtained from the 2019–2025 analysis report on China's Li‐based battery recycling industry market development status research and investment trend prospect. Global lithium, cobalt, and nickel production data are obtained from Mineral Commodity Summaries by U.S. Geological Survey.

Spent LIBs represent a precious mineral resource containing battery‐grade materials and a high content of valuable metal element components (e.g., cobalt, nickel, lithium species, etc.), and the metal element contained in the spent LIBs exceeds that of natural deposits (**Figure** **1**a,b).^[^ ^6^, ^8^, ^14^ ^]^ In addition, the significantly fluctuating costs of natural resources for manufacturing LIBs, especially lithium species, greatly affect the stable supply and profitability of producers (Figure 1c).^[^ ^9^, ^14^ ^]^ Through proper techniques, these resources from spent LIBs can be extracted and reused in the manufacturing of new batteries, establishing a closed‐loop and stable supply chain and bringing certain economic value due to the lower and more stable raw material cost.^[^ ^8^, ^9^ ^]^

### 2.2 Environmental Issues Related to Spent LIBs

The huge growth in the demand for LIBs is causing the generation of massive quantities of retired batteries each year. However, only a fraction of spent batteries are recycled, and landfill and illegal disposal remain the main methods for disposing of spent batteries.^[^ ^10^ ^]^ Spent LIBs are considered hazardous wastes because they contain heavy metal elements (e.g., Co, Ni, and Mn, classified as carcinogenic materials) and toxic organic electrolytes (which react with water to release noxious HF gas).^[^ ^9^, ^15^ ^]^ If spent LIBs are disposed of inappropriately, the wastes generated will induce adverse environmental and human health concerns and even cause safety issues such as fire and explosion.^[^ ^16^ ^]^ Therefore, from this aspect, LIBs recycling is highly desired.

### 2.3 Sustainability of Mineral Mining for LIB Fabrication

Currently, fossil fuel still accounts for a large proportion of global energy consumption, and its annual production is still in a phase of rapid growth (Figure 1d).^[^ ^6^ ^]^ However, the tremendous issues associated with nonrenewable fossil fuels require the establishment of a sustainable and environmentally friendly society where renewable energy plays the key role. In this context, LIBs have been widely used as advanced storage devices designed to store renewable energy. While the energy stored inside a LIB might be renewable, the LIBs fabrication process involving mass mineral mining is not renewable at all. The surge in demand for LIBs indicates that there will be an exponential growth in demand for mineral mining of lithium, cobalt, and other critical materials (Figure 1e).^[^ ^8^, ^9^, ^10^ ^]^ If spent LIBs cannot be recycled effectively, we will continue to consume natural resources in an unsustainable way. There will be no difference between the mining/consumption of Li/Co/Ni and the mining/consumption of coal/oil/natural gas. Thus, the key role of LIBs in the renewable energy field will be challenged.^[^ ^17^ ^]^ Unfortunately, compared with the high number of LIBs on the global market, the actual recovery rate of spent LIBs is low, less than 5% in 2019.^[^ ^18^ ^]^ China, currently the largest manufacturer and consumer worldwide of LIBs, has still low level of recovery rate, which urgently needs to be improved (Figure 1f).

### 2.4 Uneven Resource Distribution and Production

The production of major raw materials for LIBs is concentrated in a few countries, where 70% of lithium production is controlled by Australia and Chile, and 70% of cobalt production is controlled by Congo (Figure 1g–i).^[^ ^14^ ^]^ Due to the limited natural resources and geographically concentrated areas of distribution and production, relatively valuable metals such as Li, Co, and Ni from spent LIBs are considered a valuable recycling waste stream.^[^ ^6^, ^8^, ^14^ ^]^ Compared with primary production (mineral mining) of metal resources, secondary production (recycling metal resources from spent LIBs) depends less on geographical factors^[^ ^19^, ^20^, ^21^ ^]^ and therefore avoids a fragile supply chain and unstable raw material cost.^[^ ^9^, ^14^ ^]^

## 3 Recycling Strategies for Conventional LIBs

**Figure 2:**
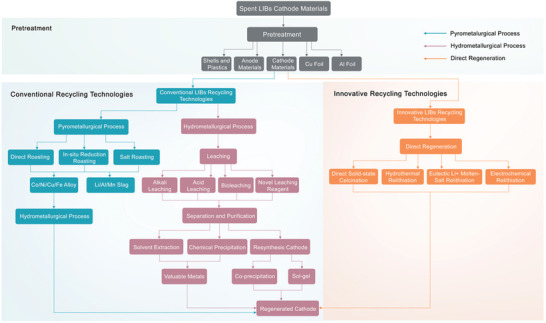
Current LIBs recycling technologies.

The recycling of spent LIBs is indispensable in terms of critical raw material supply, environmental conservation, and sustainability. The development of novel LIBs recycling technologies is currently one of the most widely researched topics in industry and academia. In this section, we present current LIBs recycling technologies, including pretreatment, conventional pyrometallurgical and hydrometallurgical recycling technologies, and innovative direct repair recycling technologies (**Figure** **2**).

### 3.1 Pretreatment

**Figure 3:**
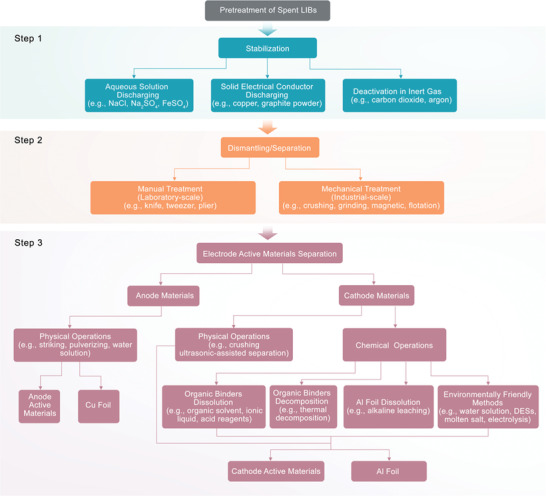
Pretreatment processes of spent LIBs.

Pretreatment is the first step of LIBs recycling and is an essential process to improve the recovery rate of valuable elements and reduce the energy consumption of subsequent steps. To avoid the safety hazards of spent LIBs, such as fire and explosion, safe and effective separation of the multiple components in LIBs is the major goal of pretreatment.^[^ ^22^, ^23^ ^]^ Pretreatment can be divided into three main processes: stabilization, dismantling/separation, and electrode active material separation, which can be carried out independently or simultaneously (**Figure** **3**).

#### 3.1.1 Stabilization

To minimize the safety risks from residual power, the stabilization of spent LIBs is essential and can be achieved by aqueous solution discharging (e.g., NaCl, Na_2_SO_4_, and FeSO_4_),^[^ ^24^, ^25^, ^26^, ^27^ ^]^ solid electrical conductor discharging (e.g., copper and graphite powder)^[^ ^24^, ^28^, ^29^ ^]^ and battery deactivation in an inert gas (e.g., carbon dioxide or argon).^[^ ^19^ ^]^ Discharging treatment is usually applied in laboratories, where NaCl salt solution is the most commonly used reagent, but there are some problems, such as Cl_2_ emission, galvanic corrosion, and electrolyte leakage.^[^ ^30^ ^]^ Solid electrical conductor discharging is used to discharge spent LIBs by mixing batteries with copper or graphite powder, but this process generates a large amount of heat inside the battery, which increases the risk of explosion.^[^ ^28^ ^]^ Currently, some recycling companies (e.g., Recupyl) skip the discharging process and instead directly dismantle/separate spent LIBs under an inert atmosphere.^[^ ^19^, ^31^ ^]^ Compared with discharging treatment, direct dismantling/separation under an inert atmosphere has less environmental pollution, lower energy consumption, and lower cost, so the latter techniques are widely used in industrial processing. However, this direct dismantling/separation process involves cross‐contamination and the loss of a significant fraction of lithium in the electrolyte and anode. Recently, the direct dismantling/separating of charged jellyroll LIBs in water (which acts as a fire extinguishing agent and oxygen isolator) was demonstrated, which enables precise nondestructive separation of LIBs components (e.g., cathode and anode materials, Cu foil, separator, and shell) and facilitates lithium recovery from the anode and electrolyte with near‐perfect recycling efficiency and no harmful emissions.^[^ ^32^, ^33^ ^]^

#### 3.1.2 Dismantling/Separation

After stabilization of spent LIBs, a dismantling/separation step is required to separate the multiple components of the batteries and collect the enriched cathode materials. This step is generally divided into two major categories: manual and mechanical treatment. Manual treatment of spent LIBs is commonly performed in laboratories, in which knives, tweezers, or pliers are used to recover different components, such as shells, separators, and anode and cathode materials. Although manual treatment minimizes impurities in the subsequent process and produces purer separated materials, it is not suitable for industrial application. In industry, mechanical treatment is superior to manual treatment. Industrial treatment can directly passivate spent LIBs in an inert atmosphere (e.g., carbon dioxide or argon) to achieve the comminution of batteries in various charged states and reduce the cost of stabilization before comminution.^[^ ^19^ ^]^ The mechanism of mechanical treatment is based on the different physical properties (e.g., size, density, wettability, and magnetism) of the cathode materials, anode materials, stainless steel, current collectors, separators, and plastics, where battery components can be easily separated by comminution (e.g., crushing and grinding)^[^ ^24^, ^34^, ^35^, ^36^, ^37^, ^38^ ^]^ and separation (e.g., size, density, magnetic or eddy current separation and froth flotation).^[^ ^34^, ^35^, ^39^, ^40^, ^41^, ^42^, ^43^, ^44^ ^]^

#### 3.1.3 Electrode Active Materials Separation

Commonly, electrode materials are attached to current collectors (Cu or Al foil); thus, exploring the separation of high‐purity active materials from current collectors is a critical prerequisite for the subsequent recycling process. Due to the weak bonding strength of graphite to Cu foil, anode materials can be easily separated from Cu foil by physical operations such as striking, pulverizing, and treatment with a water solution.^[^ ^23^, ^32^, ^45^ ^]^ The disassembly of charged jellyroll LIBs in water enables precise non‐destructive separation of graphite and Cu foil.^[^ ^32^ ^]^ For cathode materials, a series of separation methods have been developed. The cathode materials are tightly adhered to the Al foil by a binder (e.g., poly(vinylidene difluoride)), making it difficult to directly peel off the active materials from the foil. Commonly used separation processes can be divided into two categories, chemical, and physical techniques, where chemical techniques include organic solvent (e.g., *N*‐methyl‐2‐pyrrolidone (NMP), *N*,*N*‐Dimethylformamide, *N*,*N*‐Dimethylacetamide, dimethyl sulfoxide, acetone) treatment,^[^ ^46^, ^47^, ^48^, ^49^ ^]^ ionic liquid treatment,^[^ ^50^ ^]^ treatment with acid reagents to dissolve organic binders,^[^ ^51^, ^52^, ^53^ ^]^ alkaline leaching of Al foil^[^ ^54^, ^55^ ^]^ and thermal decomposition of organic binders;^[^ ^56^, ^57^, ^58^, ^59^ ^]^ physical techniques include mechanical crushing^[^ ^60^, ^61^ ^]^ and ultrasonic‐assisted separation.^[^ ^46^, ^51^ ^]^ Considering the high toxicity and expense of organic solvents and ionic liquids, the corrosiveness of acid and alkaline reagents, harmful gas emissions, and the high energy consumption of thermal treatment, some relatively green and environmental friendly processes have been developed for exfoliating cathode materials from Al foil, such as separation by water solution,^[^ ^62^, ^63^ ^]^ deep eutectic solvents (DESs),^[^ ^64^, ^65^ ^]^ molten salt,^[^ ^66^ ^]^ and electrolysis.^[^ ^67^ ^]^ In addition, the efficiency of peeling off cathode materials from Al foil can be improved with mechanical assistance.^[^ ^46^, ^51^, ^68^, ^69^ ^]^

After these processes, the electrode materials are separated and purified for subsequent processing. Although mechanical treatment plays a dominant role in industrial LIBs recycling, the inevitable interpenetration of different components remains troublesome.^[^ ^70^, ^71^ ^]^ Therefore, one of the important research directions for future LIBs recycling is the precise and nondestructive separation of battery components and constituent materials in charged spent LIBs before the recycling process.

### 3.2 Conventional LIBs Recycling Technologies

Conventional LIBs recycling technologies are mainly based on inorganic chemistry, mainly including pyrometallurgical and hydrometallurgical processes, both of which involve two steps: the destruction of crystal structure of cathode materials to the atomic level and the extraction of valuable metals elements.^[^ ^6^, ^14^, ^18^ ^]^ The pyrometallurgical process is a high‐temperature smelting process that has been widely used in industry due to its simple operation and high production efficiency and is mainly deployed in Europe and North America.^[^ ^14^, ^71^ ^]^ The hydrometallurgical process involves the use of acid (or base) aqueous solutions to extract valuable metals elements; due to the high recovery rate, low energy consumption, and relatively low harmful gas emissions, this process is widely used in industry, mainly in China.^[^ ^14^ ^]^

#### 3.2.1 Pyrometallurgical Process

The pyrometallurgical process involves pyrolysis treatment of spent LIBs. The pyrolysis products consist of valuable metals or compounds, slag, and gases; the combustion of anodes and organic materials can provide energy for this process but release toxic gases. The reducing agents in this process can reduce metal oxides to alloys (e.g., Co, Ni, Fe, Cu), and then pure metal materials can be recovered by hydrometallurgical processes, while metals such as Al, Li, and Mn remain in the slag. As the value of lithium increases, the recycling of lithium is becoming greatly important, thus the Li in the slag requires to be recycled. The most prominent advantage of the pyrometallurgical process is that the whole cell or module can be handled directly, so this process is widely used in industry. This technology can be divided into three major categories: direct roasting, in situ reduction roasting, and salt roasting.^[^ ^8^, ^14^, ^72^ ^]^

##### Direct Roasting

Direct roasting is the early pyrometallurgical process, its principle is to reduce metal oxides into alloys at high temperature (>1000 °C) through reducing agents (e.g., carbon, aluminum).^[^ ^72^, ^73^, ^74^ ^]^ Subsequently, pure metal materials can be recovered from alloys by hydrometallurgical process. In the typical pyrometallurgical process, first, spent LIBs and reducing agents are calcined in a high‐temperature furnace; then, slag‐forming agents (e.g., CaO, Al_2_O_3_, SiO_2_) are introduced into furnace to separate alloys from slags.^[^ ^74^, ^75^, ^76^ ^]^ In this process, anode material, Al current collectors, and organic materials (e.g., electrolyte, plastic, separator, binder) from spent LIBs can be utilized as reducing agents or fuels, reducing the cost and energy consumption.^[^ ^8^, ^14^ ^]^ However, metals such as Al, Li, and Mn remain in slag, causing waste of resources and environmental pollution, especially considering the increased value of lithium. Many approaches have been explored to recover lithium from slag, such as introducing CaCl_2_ or Na_2_SO_4_ into slag for roasting to realize the conversion of insoluble lithium to water‐soluble lithium salt.^[^ ^77^, ^78^, ^79^, ^80^ ^]^ This direct roasting technology has relatively low requirements for spent LIBs, which is beneficial to the large‐scale industrial processing of spent batteries. However, direct roasting has high energy consumption, serious environmental pollution, and a low recovery rate, which has prompted the development of novel processes such as in situ reduction roasting and salt roasting.^[^ ^6^, ^72^, ^81^, ^82^ ^]^

##### In Situ Reduction Roasting

**Figure 4:**
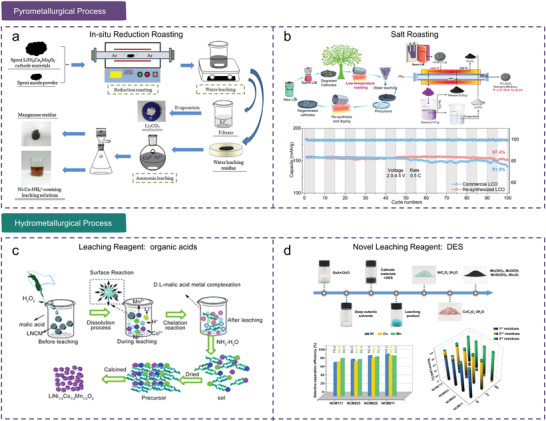
Conventional LIBs recycling technologies. a) Schematic of the recovery of valuable metals from spent LIBs by in situ reduction roasting. Reproduced with permission.^[^ ^88^ ^]^ Copyright 2021, Elsevier. b) Top left: schematic of the closed‐loop recycling process for the conversion of spent LiCoO_2_ to high‐voltage LiCoO_2_. Top left: schematic of the entire recycling process of spent LiCoO_2_ using (NH_4_)_2_SO_4_ salt. Bottom: cycling performance of resynthesized and commercial LiCoO_2_ at 4.5 V. Reproduced with permission.^[^ ^33^ ^]^ Copyright 2021, Elsevier. c) Schematic of spent NCM cathode recycling using malic acid. Reproduced with permission.^[^ ^104^ ^]^ Copyright 2016, RSC. d) Top: flowchart of the selective recovery of valuable metals from NCM cathode using DES. Bottom left: selective separation efficiencies of different NCM cathodes. Bottom right: recovery yields of different NCM cathodes. Reproduced with permission.^[^ ^105^ ^]^ Copyright 2022, Wiley‐VCH.

In situ reduction roasting refers to the conversion of spent LIBs into high‐value products (e.g., metal oxide, pure metal, soluble lithium salt) under a vacuum or inert atmosphere via pyrolysis without adding other additives, which is different from direct roasting. In situ reduction roasting reduces the conversion temperature of cathode materials, simplifies the procedures, and reduces the recovery cost, and it is being studied at the laboratory scale. Xu et al. first reported the recovery of Co, Li, and graphite from spent LiCoO_2_‐based LIBs by in situ reduction roasting under an N_2_ atmosphere at 1000 °C for 0.5 h using graphite as a reducing agent.^[^ ^83^ ^]^ However, the roasting temperature over 750 °C can easily cause the volatilization of Li_2_CO_3_ and lead to a significant mass loss (≈30%).^[^ ^84^, ^85^ ^]^ To further reduce the roasting temperature, they proposed replacing the inert atmosphere with a vacuum to further move the thermodynamic equilibrium of the carbothermal reaction toward low temperature (<800 °C).^[^ ^86^, ^87^ ^]^ Zhou et al. recycled Li (82.2%), Co (99.1%), and Ni (97.7%) from spent NCM (LiNi_1−_ *_y_*_−_ *_z_* Co*_y_* Mn*_z_* O_2_) cathode using in situ reduction roasting combined with water and ammonia leaching (**Figure** **4**a).^[^ ^88^ ^]^ Since these pioneering works, Xu et al. proposed a theoretical analysis of the in situ carbothermal reduction roasting and established a collapsing model of spent LIBs pyrolysis.^[^ ^86^, ^89^ ^]^ From the crystal structure, graphite has a stronger affinity for O atoms than Li or Co atoms, resulting in the instability and collapse of LiCoO_2_ oxygen octahedrons (also suitable for cathode materials with similar oxygen octahedrons, such as NCM, LiMn_2_O_4_), where Li atoms are released in the form of Li_2_CO_3_ and O atoms are captured by graphite to form CO_2_. In this process, graphite can promote the decomposition of cathode materials due to the coupling reaction of the graphite anode burning and cathode pyrolysis.^[^ ^86^, ^88^, ^89^ ^]^ However, before carbothermic reduction roasting, it is necessary to separate cathode materials from Al foil, which increases the complexity of the recycling process. In contrast to carbothermic reduction roasting, Al current collector within spent LIBs is used as a thermal reducing agent in thermite reduction roasting, which presents the following advantages: cathode materials do not need to be peeled off from Al foil; a lower roasting temperature and lower energy consumption can be achieved because of more active chemical properties of Al than carbon; and the harmful gases released by carbothermic reduction roasting, such as CO_2_ and CO, are avoided.^[^ ^82^ ^]^ Therefore, using Al foil as a reducing agent is simpler and cleaner. Based on these prominent advantages, Xu et al. reported the recovery of Co, Li, and Al from spent LiCoO_2_‐based LIBs at 600 °C for 1 h under Ar atmosphere with Al foil as a reducing agent.^[^ ^90^ ^]^ Furthermore, according to thermodynamic analysis, LiCoO_2_ first reacts with Al rather than carbon, and is directly reduced to CoO because of the instability of the Co (III) at high temperature. The final roasting products of spent LiCoO_2_ are CoO, Li_2_O, and LiAlO_2_, which are then selectively leached out by alkaline and acid leaching. Recently, they demonstrated that the method is also applicable to spent NCM‐based LIBs, indicating the wide applicability of in situ thermite reduction roasting.^[^ ^82^ ^]^ Both in situ carbothermal and thermite reduction roasting can simplify procedures, lower energy consumption, and selectively recover lithium; nevertheless, the exhaust gas released from carbothermal roasting requires secondary treatment, and waste effluent from thermite roasting needs to be treated.

##### Salt Roasting

To further reduce the roasting temperature and increase the metal recovery rate, various classes of salt roasting technologies have been proposed to recycle spent LIBs, such as chlorination roasting, sulfation roasting, and nitration roasting.^[^ ^91^, ^92^, ^93^ ^]^ Salt roasting has attracted great interest in pyrometallurgical processes because of the prominent advantages, such as high reactivity, high volatility, high solubility, a low melting point, and the environmental friendliness of salts as well as low‐cost preparation techniques and simple operation.^[^ ^94^, ^95^ ^]^ The essence of salt roasting is that the crystal structure of cathode materials can be easily destroyed at low temperature, and the components can be completely converted into water‐soluble salts, thus greatly improving the overall metal (especially lithium) recovery efficiency and reducing the waste effluent.^[^ ^6^, ^72^ ^]^ A related technology for metal recovery from spent LIBs, sulfation roasting (≈600 °C) based on acidic sulfates (e.g., MgSO_4_, NaHSO_4_, and Na_2_SO_4_), has been developed, where LCO (LiCoO_2_) is converted into soluble lithium salts (e.g., Li_2_SO_4_ and LiNaSO_4_) and insoluble transition metal oxides (e.g., CoO and Co_3_O_4_).^[^ ^96^, ^97^ ^]^ Recently, our group proposed a closed‐loop and environmentally friendly recycling technology for converting spent LiCoO_2_ into high‐voltage LiCoO_2_ cathodes based on low‐temperature roasting (<400 °C, with the assistance of (NH_4_)_2_SO_4_) combined with water leaching (Figure 4b).^[^ ^33^ ^]^ According to previous studies, chlorination roasting (≥900 °C with solid chlorinating agents, such as NaCl, CaCl_2_, and MgCl_2_) is widely used to extract metals (e.g., Ni, Co, and Li) from minerals or concentrates.^[^ ^95^, ^98^, ^99^, ^100^ ^]^ Subsequently, Sun et al. proposed the use of CaCl_2_ as a chlorine donor to recover lithium from slag after conventional pyrometallurgical treatment of spent LIBs.^[^ ^101^ ^]^ Although these works have shown that salt‐assisted roasting is feasible for the recovery of spent LIBs, the high roasting temperature remains a troublesome obstacle. NH_4_Cl is a widely available, nontoxic, and noncorrosive solid chlorination and reducing agent, and its introduction into roasting can not only destroy the crystal structure of cathode materials (e.g., LiCoO_2_, LiMn_2_O_4_ and LiCo_1/3_Mn_1/3_Ni_1/3_O_2_) at a low temperature (<600 °C) but also reduce the valence state of transition metals, such as Co ions in LCO, which are reduced to Co^2+^.^[^ ^94^ ^]^ The formed highly soluble chloride salts (e.g., CoCl_2_ and LiCl) can be recovered by water leaching.^[^ ^81^, ^94^ ^]^ In this process, NH_4_Cl is used as the only additive without the addition of any strong acid or reducing agent, and the redundant NH_4_Cl can be recycled; thus, NH_4_Cl‐based roasting is considered an environmentally friendly method.^[^ ^102^ ^]^ Nitration roasting is another salt roasting technology with low roasting temperature and good selectivity, where transition metals from spent LIBs are first converted into nitrates and then decomposed into insoluble oxides (<250 °C), while lithium remains in a salt state (LiNO_3_) due to its high decomposition temperature (≈600 °C).^[^ ^72^, ^92^, ^103^ ^]^ In the subsequent water‐leaching process, compared with other lithium salts (e.g., Li_2_CO_3_, LiCl, and Li_2_SO_4_), LiNO_3_ has higher solubility, which can effectively improve the recovery rate of lithium salts.^[^ ^92^ ^]^ Furthermore, the temperature of nitration roasting is relatively low, reducing the costs. Salt‐assisted roasting can destroy the crystal structure of the cathode at low temperatures, thereby improving the metal recovery rate of spent LIBs, and thus presents great potential for industrial application. However, salt roasting technology still faces some challenges, such as the emission of toxic and harmful gases (e.g., SO*_x_*, Cl_2_, and NO*_x_*), and the roasting temperature needs to be further reduced.^[^ ^72^ ^]^

Pyrometallurgical process, with simple operation, high productivity, and mature technology, has achieved relative success in portable electronic device batteries with a high cobalt content, while the low cobalt content or cobalt‐free nature of LIBs for electric vehicles makes this business model less appealing. Future pyrometallurgical processes should focus on the development of low‐temperature roasting closed‐loop recycling technologies that can be applied to low‐cobalt and cobalt‐free cathode materials.

#### 3.2.2 Hydrometallurgical Process

Hydrometallurgical process involves aqueous chemical treatment of spent LIBs with two steps: leaching and separation; the leaching step plays a decisive role in the metal dissolution efficiency of spent LIBs, while the separation and purification step play a decisive role in metal separation, impurity removal, and the production of high‐purity materials.^[^ ^106^ ^]^ In this process, acids, alkali, or special solvents (e.g., DESs and molten salts) are used as leaching media to dissolve the desired metals (e.g., Li, Co, Ni, and Mn) into the solution. Then, through a series of separation technologies (e.g., solvent extraction, chemical precipitation, and sol‐gel techniques), a high recovery rate of metals is achieved, and new cathode materials or other high value‐added products are generated.^[^ ^8^ ^]^ Compared with pyrometallurgical process, hydrometallurgical process has significant advantages, such as low energy consumption, maximum recovery of LIBs components, high‐purity products, and lower CO_2_ emissions. However, the current hydrometallurgical process for spent LIBs recycling is inadequate due to factors such as the need for sorting, difficult separation for the similar properties of metal ions (e.g., Co, Ni, Mn, Cu, and Al) in solution, and the high cost of wastewater treatment.^[^ ^14^ ^]^ In this section, we provide a comprehensive overview of various technical improvements in leaching and separation of spent LIBs.

##### Step 1: Leaching

As the first step of hydrometallurgical process, leaching can dissolve valuable metals in spent LIBs into a solution to prepare for subsequent separation and purification. Leaching can be divided into four major categories: alkali leaching, acid leaching, bioleaching, and special solvent leaching.

Alkali leaching: Alkali leaching has attracted great interest for recycling desired metals from spent LIBs due to its selectivity and low‐cost separation; in this technique, the leaching system generally consists of two parts: ammonia (NH_3_) and a reducing agent (e.g., Na_2_SO_3_, (NH_4_)_2_SO_3_ or H_2_O_2_).^[^ ^8^, ^14^, ^107^ ^]^ The selective leaching mechanism of ammonia‐based systems is that the chelation between NH_3_ and certain metal ions (e.g., Li, Ni, Co, and Cu; Al, Mn, and Fe have weak complexing ability) forms stable and water‐soluble ammonia complexes.^[^ ^88^, ^108^, ^109^ ^]^ The introduction of reducing agents can reduce the oxidation state of transition metals (e.g., Ni^3+^ and Mn^4+^) and form highly soluble salts, improving the leaching efficiency of various metals and shortening the reaction time.^[^ ^110^ ^]^ However, conventional binary systems (i.e., ammonia + reducing agent) suffer from severe pH changes that affect the formation of soluble complexing ions. Therefore, buffers were introduced into the above system to obtain a ternary system (i.e., ammonia + reducing agent + buffer), and a stable pH environment was constructed in which the pH was determined by the base (NH_3_) and the conjugate acid (NH_4_^+^ from the buffer).^[^ ^111^ ^]^ However, due to the weak complexing ability of Mn and ammonia, the obtained Mn salts contain many impurities, which cannot be recycled as valuable products. Therefore, achieving high‐efficiency leaching and obtaining high‐purity Mn salt is still a significant challenge for the recovery of spent LIBs by alkali leaching systems. In addition, recently, Kang et al. proposed green method for recycling all elements (e.g., Li, Fe. P) from spent LiFePO_4_ batteries by water and NaOH solution at room temperature.^[^ ^112^ ^]^

Acid leaching: Acid leaching has attracted the most attention because of its high leaching efficiency and low cost and can generally be divided into two categories: inorganic acid leaching and organic acid leaching. In contrast to alkali leaching, the acid leaching method can dissolve almost all the metals in spent LIBs into the leaching solution. Inorganic acid leaching reagents include HCl,^[^ ^113^, ^114^ ^]^ HNO_3_,^[^ ^115^, ^116^ ^]^ H_2_SO_4_,^[^ ^117^, ^118^, ^119^ ^]^ and H_3_PO_4_,^[^ ^120^, ^121^ ^]^ and these reagents may cause secondary pollution (emission of hazardous gases, such as Cl_2_, NO*_x_* and SO*_x_*, and the production of a large amount of acidic wastewater) during the process, increasing environmental and safety risks.^[^ ^122^ ^]^ Reducing agents (e.g., H_2_O_2_ and NaHSO_3_) are usually added to inorganic acid leaching systems to reduce the valence state of metal ions (e.g., Ni^3+^ and Mn^4+^) and accelerate the dissolution rate of metal ions.^[^ ^119^, ^123^ ^]^ Commonly used organic acid leaching reagents, such as citric acid,^[^ ^124^ ^]^ malic acid,^[^ ^104^, ^125^ ^]^ and oxalic acid^[^ ^126^, ^127^, ^128^, ^129^ ^]^ can offer similar leaching efficiency (mainly determined by their acidity^[^ ^6^, ^68^ ^]^) to that of inorganic acid leaching in a mild environment and are biodegradable. In addition, some organic acids have unique properties, such as chelating coordination (e.g., citric acid and malic acid),^[^ ^54^, ^104^, ^130^ ^]^ which can recover metal ions from leaching solution to regenerate new cathode materials, and reduction (e.g., ascorbic acid and oxalic acid),^[^ ^127^, ^131^, ^132^ ^]^ which can eliminate the need to add reducing agents during acid leaching. Xi et al. used malic acid as both a leaching reagent and chelating agent for recycling valuable metal ions from spent NCM, and new NCM cathodes were synthesized by a sol‐gel process (Figure 4c).^[^ ^104^ ^]^ In addition, due to the different solubilities of oxalates, oxalic acid can perform selective leaching; only lithium oxalate can be dissolved in the leaching solution, and other transition metal oxalates are precipitates.^[^ ^127^, ^132^ ^]^ Typical acid leaching usually requires excess and high concentrations of acid to ensure a high leaching efficiency but also produces a large amount of acidic wastewater. To reduce the amount of acid and improve the leaching efficiency, electrochemical methods,^[^ ^133^, ^134^ ^]^ ultrasonic treatment^[^ ^114^, ^135^ ^]^ and mechanochemical methods^[^ ^132^ ^]^ have been introduced into the acid leaching process. Taking the mechanical method as an example, the electrode material is first ground with a co‐grinding reagent to achieve the easy leaching of metal ions or the formation of soluble chelates; second, metal ion leaching is carried out with water leaching or acid leaching.^[^ ^132^, ^136^ ^]^

Bioleaching: Bioleaching is an emerging technology for metal extraction from spent LIBs and is complementary to alkali and acid leaching.^[^ ^137^, ^138^, ^139^ ^]^ Compared with alkali and acid leaching, bioleaching is an environmentally friendly method that utilizes inorganic (e.g., H_2_SO_4_) and organic acids (e.g., oxalic acid, citric acid, and malic acid) produced by bacteria (e.g., *Acidithiobacillus ferrooxidans*)^[^ ^140^, ^141^ ^]^ and fungi (e.g., *Aspergillus niger*),^[^ ^137^, ^138^, ^142^, ^143^ ^]^ respectively, during metabolization to leach spent LIBs. Although bioleaching has prominent advantages such as environmental friendliness and energy savings, the long microbial culturing time, susceptibility to contamination and low leaching efficiency limit its industrial application, the challenges it faces can potentially be addressed through further research.

Novel leaching reagent: Based on the drawbacks of traditional alkali/acid leaching (e.g., secondary pollution) and bioleaching (e.g., low leaching efficiency), researchers shifted their focus to novel leaching reagents (e.g., DESs and supercritical fluids (SCFs)).^[^ ^8^, ^144^ ^]^ DES is an environmentally friendly solvent, a eutectic mixture formed by two or more nontoxic compounds through hydrogen bonding; its melting point is lower than that of the individual components, and it has a great ability to dissolve metal oxides.^[^ ^145^ ^]^ Ajayan et al.^[^ ^64^ ^]^ first proposed the use of a DES (choline chloride + ethylene glycol) for spent LIBs recycling (including LiCoO_2_ and NCM systems). A DES is an effective leaching and reducing agent that achieves the extraction of metals and the separation of other battery components, such as Al foil and binder. Since the leaching efficiency is related to the composition of the DES, the stronger the reducibility of the DES is, the higher the leaching efficiency of high‐valent metals, so the priority is to screen suitable DESs for metal leaching. Subsequently, Zhang et al.^[^ ^146^ ^]^ proposed a novel method for screening suitable DESs and found that DESs composed of choline chloride and urea had higher reducing power and required a lower leaching time (12 h) and temperature (180 °C); the extraction efficiency of Li and Co was 95%. Riaño et al.^[^ ^147^ ^]^ proposed another green, cheap and safe DES (choline chloride + citric acid) to recover cobalt (81%) and lithium (99.9%) from spent LIBs. Recently, Guo et al. successfully stepwise separated nickel (99.1%), cobalt (95.1%), and manganese (95.3%) from spent NCM cathode materials by regulating the coordination environment of transition metal complexes in the DES (composed of choline chloride and oxalic acid dihydrate) (Figure 4d).^[^ ^105^ ^]^ Although DESs have successfully achieved the dissolution of metal oxides, they have not been fully explored in the recycling of spent LIBs, so the development of novel DESs with high leaching efficiency and low cost is a future research direction.

Another environmentally friendly solvent is SCFs (e.g., water or methanol), which have been widely used in the disposal of waste electrical and electronic devices.^[^ ^148^, ^149^ ^]^ Compared with conventional leaching, SCFs techniques can provide an extreme environment to achieve higher metal leaching efficiency. Liu et al.^[^ ^150^ ^]^ proposed an environmentally friendly process for spent LIB recycling and simultaneous detoxification of polyvinyl chloride (PVC) in subcritical water, in which PVC dechlorination was used to provide an HCl source to leach metals, while the PVC dechlorination products were converted into nontoxic compounds. This innovative subcritical cotreatment process of PVC and spent LIBs is environmentally friendly, economic, and efficient. In addition, Bertuol et al.^[^ ^151^ ^]^ demonstrated that supercritical extraction is superior to conventional leaching methods. These results confirm the feasibility of using SCFs in the recovery of spent LIBs, but further exploration is still needed to achieve low‐cost and high‐efficiency metal leaching. In addition, Wang et al. reported an innovative recycling technology based on a redox targeting‐based process, where high‐purity LiOH (99.90%) and FePO_4_ (99.97%) were obtained from spent LiFePO_4_ via a solution of [Fe(CN)_6_]^3–^ (as a selective and regenerative redox mediator).^[^ ^152^ ^]^

##### Step 2‐1: Separation and Purification

As the second step of hydrometallurgical process, separation and purification play a decisive role in the purity and properties of the product and include solvent extraction and chemical precipitation methods.

Solvent extraction: After metal leaching, solvent extraction is carried out to remove and separate impurities (e.g., Al, Cu, and Fe) according to the solubility of metal ions in an organic‐water two‐phase system to obtain a satisfactory level of metal purity.^[^ ^6^, ^8^ ^]^ Generally, the valuable metal ions in the leaching solution are Li^+^, Ni^2+^, Co^2+^, and Mn^2+^. Commonly used solvent extractants are bis‐(2‐ethylhexyl) phosphoric acid (D2EHPA),^[^ ^153^ ^]^ 2‐ethylhexyl phosphoric acid mono‐2‐ethylhexyl ester (PC‐88A),^[^ ^154^ ^]^ (2‐ethylhexyl)(2,4,4′‐trimethylpentyl)phosphinic acid (USTB‐1)^[^ ^155^ ^]^ and bis(2,4,4‐ trimethylpentyl) phosphinic acid (Cyanex272),^[^ ^156^ ^]^ and stripping reagents mainly use H_2_SO_4_. Compared with single solvent extractants, extraction systems composed of multiple solvent extractants, such as the D2EHPA‐Cyanex272,^[^ ^157^ ^]^ D2EHPA‐PC‐88A,^[^ ^36^ ^]^ and Cyanex272‐PC‐88A^[^ ^158^ ^]^ system, have higher extraction efficiency and metal selectivity. Other extraction conditions, such as the solvent extractant concentration, organic/aqueous (O/A) ratio, pH and reaction temperature, and time, can also affect the extraction efficiency and metal selectivity.^[^ ^159^ ^]^ In addition, metal ions with similar chemical properties can be effectively separated through multistage extraction and stripping. Although solvent extraction has many advantages, such as operation at room temperature, a short reaction time (≈30 min), and high purity of products, the high cost of solvents and complicated processing technology limit its further application. Therefore, future perspectives include the development of environmentally friendly and sustainable solvent extractants and the simplification of the extraction process.

Chemical precipitation: Chemical precipitation is another method used to separate metals and remove impurities in which the precipitation of different metals is achieved by adjusting the pH value.^[^ ^6^, ^160^ ^]^ Generally, impurities such as Al, Cu, and Fe are precipitated at a low pH, so impurities are first removed from the products. In the further recovery process, according to the different physical properties of Li ions and transition metal ions (e.g., Ni, Co, and Mn), the latter are usually precipitated first, followed by Li^+^.^[^ ^6^, ^161^ ^]^ Commonly used transition metal ion precipitants are sodium hydroxide (NaOH),^[^ ^162^ ^]^ sodium carbonate (Na_2_CO_3_),^[^ ^161^ ^]^ sodium sulfide (Na_2_S),^[^ ^163^ ^]^ oxalic acid (H_2_C_2_O_4_)^[^ ^164^ ^]^ and ammonium oxalate ((NH_4_)_2_C_2_O_4_),^[^ ^165^ ^]^ which form insoluble transition metal hydroxides or salts. Subsequently, lithium ions are separated from the leaching solution using lithium precipitants, mainly sodium carbonate (Na_2_CO_3_),^[^ ^166^ ^]^ sodium phosphate (Na_3_PO_4_),^[^ ^167^ ^]^ and phosphate (H_3_PO_4_),^[^ ^168^ ^]^ to form the corresponding insoluble lithium salts. However, sequential separation is difficult due to the similar properties of transition metals. Using selective precipitation instead of conventional chemical precipitation is a promising method to overcome the above problems; for example, sodium hypochlorite (NaClO) selectively precipitates Co^2+^ to Co_2_O_3_·3H_2_O,^[^ ^169^ ^]^ potassium permanganate (KMnO_4_) selectively precipitates Mn^2+^ to MnO_2_,^[^ ^170^ ^]^ and dimethylglyoxime (DMG) selectively precipitates Ni^2+^ to nickel dimethylglyoxime chelate (Ni‐DMG).^[^ ^171^ ^]^

Other methods for metal separation include adsorption, such as the use of mesoporous γ‐Al_2_O_3_ monoliths to extract and recover Co^2+^;^[^ ^172^ ^]^ electrodeposition, such as the use of two electrodes to recover spent LiFePO_4_ and LiCoO_2_ to Fe_2_O_3_/CoPi (cobalt phosphate‐based catalyst, CoPi) photoanodes through a direct reduction mechanism for water oxidation;^[^ ^173^ ^]^ and electrolysis,^[^ ^174^, ^175^ ^]^ such as the recovery of Li and FePO_4_/C from LiFePO_4_ cathodes using an anionic membrane slurry.^[^ ^174^ ^]^ However, due to the disadvantages of high energy consumption and high cost, it is difficult to apply electrodeposition and electrolysis to the recovery of spent LIBs on a large scale. Therefore, it is necessary to explore metal separation methods with low energy consumption, low cost, environmental friendliness, and high efficiency in the future.

##### Step 2‐2: Resynthesis

For Ni, Co, and Mn, which have similar properties, it is difficult to achieve effective separation by solvent extraction or chemical precipitation. Compared with traditional separation methods, resynthesis of cathode materials has been recognized as a promising method for spent LIBs recycling. This technique not only maximizes the recovery of cathode materials but also avoids complicated separation processes and reduces the recycling cost and energy consumption. Commonly used leaching‐resynthesis methods can be divided into coprecipitation and sol‐gel methods; that is, metal ions in leaching solution (mainly acid leaching solution) are resynthesized into cathode materials.^[^ ^6^, ^8^ ^]^

Co‐precipitation: The mechanism of co‐precipitation is as follows. By adjusting the proportion of metal ions in the leaching solution, co‐precipitants are introduced to form cathode material precursors, mixed with a certain lithium salt, and calcined to obtain the regenerated cathode material. This method is not suitable for organic acid leaching systems because the complexation of organic acids will change the precipitation properties of transition metal ions, resulting in the impossibility of regenerating the coprecipitations. However, this problem does not exist in inorganic acid leaching systems.^[^ ^119^, ^176^ ^]^ Commonly used co‐precipitants are mainly hydroxide^[^ ^119^, ^176^, ^177^ ^]^ or carbonate^[^ ^178^ ^]^ system. Sun et al. regenerated Mn‐rich cathode materials from a leaching solution of mixed‐type spent cathode materials (LiMn_2_O_4_, LiCoO_2_, and LiNi*_x_* Co*_y_* Mn_1‐_ *_x_*_‐_ *_y_* O_2_).^[^ ^179^ ^]^ Xu et al. proposed a facile co‐extraction and co‐precipitation method to regenerate cathode materials (LiNi_1/3_Co_1/3_Mn_1/3_O_2_), which exhibited good electrochemical performance.^[^ ^180^ ^]^ Co‐precipitation method can achieve homogeneous mixing of transition metals at the molecular level and has good application prospects.

Sol‐gel: Sol‐gel treatment is another method to regenerate cathode materials without a complicated separation process.^[^ ^181^ ^]^ In contrast to co‐precipitation, sol‐gel method is suitable for both organic (e.g., malic acid, lactic acid, and citric acid)^[^ ^54^, ^181^, ^182^, ^183^ ^]^ and inorganic acid leaching systems, but it is better for the former system because organic acids can act as both leaching and chelating agents, while the latter system requires additional chelating agents. In addition, compared with co‐precipitation method, sol‐gel method can achieve the uniform mixing of all metal ions (including Li^+^) at the molecular level so that resynthesized cathode materials with uniform particles, high purity, and better electrochemical performance can be obtained.^[^ ^8^ ^]^ The operation steps of sol‐gel method are as follows. First, the ratio of metal ions in the leaching solution is adjusted to the desired value; second, the sol is transformed into a gel state; and finally, the resynthesis of the cathode material is realized by drying and sintering.^[^ ^14^ ^]^ The combination of organic acid leaching and sol‐gel method to resynthesize cathode materials does not require additional chelating agents, and no toxic gas or acidic wastewater is generated.

In this section, we have reviewed the recent progress in conventional LIBs recycling technologies, including pyrometallurgical and hydrometallurgical processes. Both methods have certain drawbacks; therefore, great efforts are still needed to develop novel recycling methods with high efficiency, environmental friendliness, and unique application benefits.

### 3.3 Innovative LIBs Recycling Technologies

**Figure 5:**
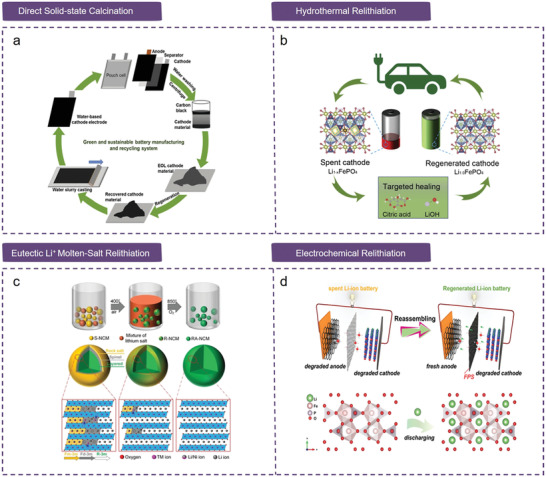
Innovative LIBs recycling technologies. a) Schematic of direct regeneration of spent LIBs NCM523 cathode. Reproduced with permission.^[^ ^63^ ^]^ Copyright 2020, Elsevier. b) Schematic of direct regeneration of spent LIBs LiFePO_4_ cathodes by targeted healing. Reproduced with permission.^[^ ^188^ ^]^ Copyright 2020, Elsevier. c) Schematic of eutectic Li^+^ molten‐salt relithiation to directly regenerate spent NCM cathode and the structure microscopic evolution. Reproduced with permission.^[^ ^187^ ^]^ Copyright 2022, Wiley‐VCH. d) Schematic of direct regeneration of spent LiFePO_4_ cathodes by electrochemical relithiation and the structural microscopic evolution. Reproduced with permission.^[^ ^189^ ^]^ Copyright 2022, Wiley‐VCH.

Innovative LIBs recycling technologies are nondestructive recycling technologies (i.e., direct regeneration/repair) that preserve the original cathode crystal structure and prevent it from being destroyed to the atomic level.^[^ ^184^, ^185^ ^]^ Compared with traditional recycling technologies, direct regeneration has undergone rapid progress because of its prominent advantages, such as a simple process, low cost, environmental friendliness, and the ability to reuse the cathode materials after regeneration (**Figure** **5**).^[^ ^186^ ^]^ The main reasons for the failure of cathode materials in LIBs can be attributed to the following: irreversible phase transformation caused by lithium loss, such as the formation of the spinel phase and rock salt phase in layered structure cathodes; and thickening of the solid‐electrolyte interface (SEI) layer accelerating the loss of lithium.^[^ ^185^ ^]^ Direct regeneration achieves the direct repair of spent cathode materials through the combination of different lithium replenishment technologies with heat treatment and includes direct solid‐state calcination, hydrothermal relithiation, and eutectic Li^+^ molten‐salt relithiation.^[^ ^63^, ^187^, ^188^ ^]^

#### 3.3.1 Direct Solid‐State Calcination

Direct solid‐state calcination is a versatile tool for regenerating cathode materials, especially single‐component LiCoO_2_, LiFePO_4_, or NCM.^[^ ^190^, ^191^, ^192^, ^193^ ^]^ Liu et al. proposed a method to directly regenerate spent LiFePO_4_ by solid‐state calcination, at an appropriate temperature, by doping with new LiFePO_4_ to regenerate the spent material.^[^ ^192^ ^]^ Zhang et al. directly regenerated spent LiFePO_4_ with Li_2_CO_3_ at 650 °C and obtained excellent physical, chemical, and electrochemical performance.^[^ ^191^ ^]^ Li et al. proposed a green manufacturing and direct recycling process, where the NMP solvent was replaced by water during electrode fabrication and spent cathodes were regenerated by direct solid‐state calcination (Figure 5a).^[^ ^63^ ^]^ In addition, Chen et al. regenerated spent NCM111 cathode materials and found that the separation method and calcination temperature had an important effect on the electrochemical performance of regenerated NCM11.^[^ ^194^ ^]^ However, since regenerated cathode materials are sensitive to impurity metals, although Cu and Al have been shown to improve the electrochemical performance of cathode materials, there is still a threshold value. Therefore, impurities need to be removed to a satisfactory purity level, which is also a major consideration for direct regeneration. Su et al. proposed and designed the introduction of Li_2_CO_3_ additives at 850 °C to directly regenerate spent LiCoO_2_ cathode materials by solid‐state calcination, and studied the influence of Cu and Al impurities on the electrochemical performance of the cathode material.^[^ ^190^ ^]^ The results showed that the influence of Cu and Al on electrode performance can be controlled within an acceptable range, and the regenerated LiCoO_2_ can meet the needs of commercial reuse. However, there are remaining challenges, including toxic exhaust emissions from binder pyrolysis and residual electrolyte, as well as poor contact between lithium source and active materials.

#### 3.3.2 Hydrothermal Relithiation

Hydrothermal relithiation is a wet chemical method and a commonly used direct regeneration method. This method can supply lithium for failed cathode materials in a low‐temperature aqueous solution, and the lithium source is uniformly distributed in the aqueous solution to realize direct contact with the cathode material. Furthermore, compared with direct solid‐state calcination, hydrothermal treatment enables the regeneration of degraded cathode materials at different states of charge without the need for precise calculation of the number of additional lithium salts.^[^ ^184^ ^]^ Chen et al. achieved the direct regeneration of cathode materials (e.g., LiCoO_2_, NCM111, and LiMn_2_O_4_) by hydrothermal relithiation in a dilute Li‐containing solution.^[^ ^184^, ^185^, ^195^ ^]^ Subsequently, they proposed a low‐temperature aqueous solution‐based direct regeneration method for the targeted repair of defects in spent LiFePO_4_ cathode materials (Figure 5b).^[^ ^188^ ^]^ Sun et al. simultaneously treated spent LIBs and vanadium‐bearing slag to achieve high‐performance regeneration of NCM111‐V_2_O_5_ cathode materials.^[^ ^196^ ^]^ In addition, Wang et al. proposed a green and efficient one‐step hydrothermal relithiation method to directly regenerate spent LiFePO_4_ cathode materials.^[^ ^197^ ^]^ The results show that hydrothermal relithiation is a flexible, simple to implement, environmentally friendly, and economical method. Further optimization of hydrothermal relithiation technology will lay the foundation for its practical application in industry.

#### 3.3.3 Eutectic Li^+^ Molten‐Salt Relithiation

Compared with solid‐state calcination and hydrothermal reactions, eutectic Li^+^ molten‐salt relithiation has been recognized as a promising technique to directly regenerate cathode materials because of the low melting point, excellent reaction medium, and fast ion diffusion.^[^ ^187^, ^198^, ^199^, ^200^ ^]^ Chen et al. first attempted the relithiation of spent NCM523 cathode materials by applying eutectic Li^+^ molten‐salt solutions (LiOH + LiNO_3_) at ambient pressure.^[^ ^200^ ^]^ Dong et al. proposed the use of another eutectic molten salt (LiOH + Li_2_CO_3_) for the direct regeneration of NCM523 cathode materials.^[^ ^199^ ^]^ Subsequently, Chen et al. proposed a ternary molten salt (LiOH + LiNO_3_ + CH_3_COOLi) system for facile, efficient direct regeneration of NCM523 cathode materials (Figure 5c).^[^ ^187^ ^]^ Recently, Dai et al. directly regenerated NCM cathodes via ionothermal synthesis using low‐cost Li halide as the Li source and ionic liquids as the solvent.^[^ ^201^ ^]^ This unique eutectic Li^+^ molten‐salt relithiation technology provides a unique platform to regenerate cathode materials at room temperature and pressure.

In addition, Gaustad et al. demonstrated that electrochemical relithiation is another method that can be applied to directly regenerate spent LiFePO_4_ cathode materials using an organic electrolyte and Li as a counter electrode.^[^ ^202^ ^]^ Subsequently, Li et al. directly regenerated LiCoO_2_ from spent LIBs by electrochemical relithiation in an aqueous electrolyte without atmospheric protection.^[^ ^203^ ^]^ Recently, Guo et al. proposed a functionalized prelithiation separator for the in situ electrochemical regeneration of degraded LiFePO_4_ cathode materials (Figure 5d).^[^ ^189^ ^]^ Since electrochemical relithiation is controlled by electrode potential, there is no need to quantify the amount of lithium deficiency prior to regeneration.

**Figure 6:**
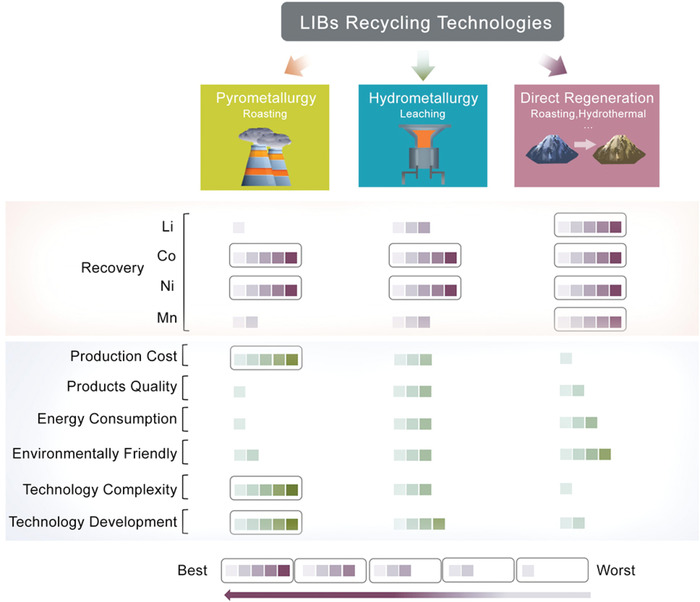
Comparison of different LIBs recycling technologies. The figure is made based on the data from ref. [19].

Although great efforts have been made to find simple and economical methods for direct regeneration, there are remaining challenges, including the need to develop appropriate pretreatment processes to avoid chemical decomposition of active materials, to guarantee a consistently high purity and pristine crystal structure of cathode materials, and to extend the technology from the lab‐scale to industry. The LIBs recycling technologies discussed are compared in **Figure** **6**.

### 3.4 Recycling of Other Battery Components

#### 3.4.1 Anode Recycling

Considering the rapid development of electric vehicles, there will be a large quantity of spent LIBs in the near future, so it is reasonable to obtain considerable economic benefits from the recycling of spent anodes.^[^ ^204^ ^]^ The main valuable components of anode recycling include graphite, copper foil, and lithium residue, where the lithium residue in the anode mainly comes from the graphite layer and SEI layer (e.g., Li_2_O, Li_2_CO_3_, LiF, ROCO_2_Li and (ROCO_2_Li)_2_) during the charging and discharging process.^[^ ^6^, ^8^ ^]^ Due to the weak bonding strength between graphite and Cu foil, Cu foil can be easily separated and recovered from the anode by physical methods such as striking and pulverizing and by aqueous solution treatment.^[^ ^23^, ^32^, ^45^ ^]^

Regarding lithium residue, since the lithium salt in the SEI layer is soluble in water, acid or water leaching can recover the lithium from the anode, while spent graphite is insoluble and retains its properties and structure after leaching. He et al. leached lithium (99.4 wt%, leaching efficiency) from the graphite anodes of spent LIBs with hydrochloric acid (HCl), and graphite with a good crystal structure was obtained after the leaching process.^[^ ^205^ ^]^ Subsequently, Bajaj et al. used water as the leaching agent to extract lithium from a graphite anode, and the lithium was recovered as solid Li_2_CO_3_ with a leaching efficiency of 99.7%.^[^ ^206^ ^]^

Regarding the recovery of graphite, previous studies mainly considered hydrometallurgical methods combined with heat treatment. Wang et al. recovered a graphite anode by a hydrometallurgical process (H_2_SO_4_ + H_2_O_2_).^[^ ^204^ ^]^ After acid leaching, the regenerated graphite was obtained by centrifugation, washing, drying, and heat treatment. Recently, Zhang et al. proposed a method to regenerate spent graphite by combining sulfuric acid curing, leaching, and heat treatment, and the impurity removal efficiency was higher than that of direct acid leaching.^[^ ^205^ ^]^ Chen and Meng et al. regenerated graphite by leaching spent graphite in a boric acid solution followed by a short heat treatment; this approach not only repaired composition and structural defects but also created functional boron doping on the graphite surface.^[^ ^206^ ^]^ In addition, due to the electrical conductivity and adsorption properties of carbon materials, spent graphite can be used to prepare other high‐value products, such as adsorbents,^[^ ^207^, ^208^, ^209^ ^]^ film,^[^ ^210^ ^]^ new anode materials,^[^ ^211^ ^]^ and graphene.^[^ ^212^, ^213^ ^]^ However, regarding the recycling of spent LIBs, there are few reports on the recycling and high‐value utilization of graphite anodes. Therefore, further exploration is needed in this research direction, which not only will be beneficial for the comprehensive recovery of LIBs but also can yield great benefits.

#### 3.4.2 Liquid Electrolyte Recycling

Electrolytes are another important source of lithium, consisting of nonaqueous organic solvents (e.g., ethylene carbonate (EC), propylene carbonate (PC), and 1,3‐dioxolane (DOL)), lithium salts (e.g., LiPF_6_, LiClO_4_, LiBF_4_, and LiTFSI) and additives.^[^ ^214^ ^]^ Part of the spent electrolyte exists in the form of a liquid, and the other part penetrates and is immobilized on the electrodes during cycling, so it is impossible to achieve a 100% electrolyte recovery rate.^[^ ^215^, ^216^ ^]^ The recycling technologies for spent electrolytes mainly include organic solvent extraction^[^ ^217^, ^218^ ^]^ and supercritical CO_2_ extraction.^[^ ^70^, ^219^ ^]^ Compared with organic solvent extraction, supercritical CO_2_ extraction has the advantages of mild operating conditions, no solvent impurities introduction, a simple purification process, and low harmful emissions. Nowak et al. first used supercritical CO_2_ to extract electrolytes from spent LIBs, and the extract contained dimethyl carbonate, ethyl methyl carbonate, EC, and trace lithium salts.^[^ ^219^ ^]^ The extract also included aging products such as diethyl carbonate, dimethyl‐2,5‐dioxahexane dicarboxylate, ethylmethyl‐2,5‐dioxahexane dicarboxylate, and diethyl‐2,5‐dioxahexane dicarboxylate. Subsequently, they optimized the supercritical and liquid CO_2_ extraction method by introducing different solvents to improve the extraction efficiency of electrolytes, especially LiPF_6_ salts. To realize closed‐loop recovery of electrolytes, Dai et al. proposed a sustainable electrolyte recycling approach, which combines supercritical CO_2_ extraction, weakly basic anion exchange resin deacidification, molecular sieve purification, and component supplementation, and the regenerated electrolyte has an ionic conductivity of 0.19 mS cm^–1^ at 20 °C.^[^ ^70^ ^]^ At present, electrolyte recovery methods mainly adopt organic solvents or supercritical CO_2_, and the high cost and difficult operation limit their further promotion. Recently, the nondestructive separation of graphite and copper foils in charged batteries in water solution was proposed and achieved lithium recovery from the electrolyte and anode.^[^ ^32^ ^]^ The future research direction is to develop green, cheap, and efficient solvents for closed‐loop recycling of electrolytes.

## 4 Recycling Strategies for Next‐Generation Li‐Based Batteries

For next‐generation batteries, there are multiple technology road maps with different properties. However, regarding battery chemistry, Li metal has always been regarded as the “holy grail” due to its lowest anode potential and high capacity. Regarding electrolytes, the evolution trend is moving from liquid‐state to all‐solid‐state electrolytes (SSEs), which strongly prioritizes high energy density and safety, and SSEs are believed to be the endpoint. Therefore, among all next‐generation battery types, all‐solid‐state Li‐metal batteries (ASSLMBs) are perceived as one of the most representative next‐generation energy storage technologies. Compared with conventional LIBs, ASSLMBs differ in structure and manufacturing procedures, mainly in the following aspects: conventional liquid electrolytes and polymer separators are replaced with SSEs (e.g., oxide‐, sulfide‐ and polymer‐based solid electrolytes); and roll‐to‐roll manufacturing processes are replaced with stacking manufacturing processes because cracks or voids may develop in the manufacturing of ASSLMBs.^[^ ^220^, ^221^, ^222^ ^]^ Failure mechanisms of ASSLMBs include electric failure, such as short‐circuit failure caused by lithium dendrites and increased interfacial resistance caused by deterioration between the electrodes and electrolytes; chemical failure, such as the reaction of highly reactive Li‐metal anodes with SSEs, which reduces battery efficiency; electrochemical failure, such as undesirable redox behaviors between electrodes and electrolytes, which influence battery performance; and mechanical failures, such as the huge volume expansion of Li‐metal anodes and cathodes, which destroy the interface structure and poor electrochemical performance.^[^ ^223^ ^]^

**Figure 7:**
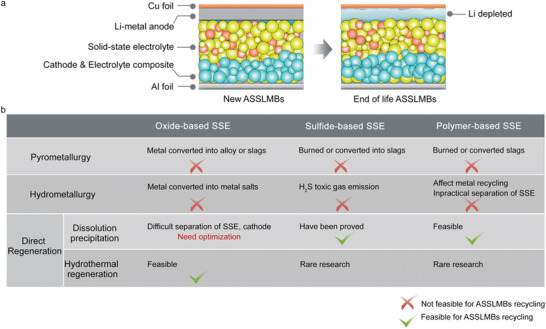
a) Schematics of components in new and end‐of‐life ASSLMBs. b) Feasibility of applying major LIBs recycling technologies to ASSLMBs.

Although some SSEs are being commercialized, the sustainability of these materials and the recycling of ASSLMBs are substantially underexplored; to date, only a few studies have focused on the recycling of sulfide‐based SSEs.^[^ ^220^ ^]^ In addition, highly reactive lithium and SSEs will bring challenges to ASSLMBs recycling, including the separation of SSEs from other battery components without damaging the electrolyte and electrode structure; the disposal of residual Li metal, where the adhesive nature of Li metal results in difficult mechanical separation and high reactivity, causing significant safety hazards; and safety hazards from SSEs, where sulfide‐based SSEs are easily hydrolyzed to generate H_2_S toxic gas in a moist atmosphere, oxide‐based SSEs form various Li salts on their surface at atmospheric, and polymer‐based SSEs and readily hygroscopic.^[^ ^220^, ^221^, ^222^, ^224^ ^]^ Based on the above issues, recycling methods used for conventional LIBs may not be suitable for ASSLMBs, and additional significant factors need to be considered. The following section discusses whether the recovery methods used for LIBs, including pretreatment methods, pyrometallurgical processes, hydrometallurgical processes, and direct regeneration, are suitable for ASSLMBs (**Figure** **7**).

### 4.1 Pretreatment

Industrial comminution technologies can directly passivate and break conventional spent LIBs under an inert atmosphere and then separate them according to the different physical properties of different components to obtain separated and purified electrode materials for subsequent processing.^[^ ^19^, ^225^ ^]^ However, for Li‐metal batteries, the strong adhesion and high reactivity of Li metal lead to difficulties in the mechanical separation (e.g., crushing and grinding) of spent ASSLMBs. In addition, SSEs are mixed thoroughly within the electrode materials except for the Li‐metal anode, which increases the contact and forms an ion transport network between the electrolyte and electrode but leads to a difficult separation between the electrolyte and electrode.^[^ ^220^ ^]^ Concerning oxide‐based SSEs, co‐sintering of SSEs and electrodes forms a dense sintered layer, it is unrealistic to physically separate the cathode material from the electrolyte, and the hardness of oxide‐based SSEs may cause damage to mechanical separation tools. Recently, Chen et al. proposed a sustainable design for fully recyclable ASSLMBs using ethanol to achieve the separation of Li_6_PS_5_Cl electrolyte from the electrodes and directly regenerated the batteries.^[^ ^226^ ^]^ Therefore, it is necessary to develop targeted methods for the pretreatment of ASSLMBs to achieve effective separation of each battery component for subsequent recycling.

### 4.2 Pyrometallurgical Process

Pyrometallurgical process, as a highly productive and mature technology, has been widely used to extract valuable metals from spent LIBs.^[^ ^19^ ^]^ Regarding ASSLMBs, sulfide‐ and polymer‐based SSEs are burned or converted into slag after pyrolysis treatment, and sulfide releases toxic gases; metal elements (e.g., La, Zr, Ti, and Ge) in oxide‐based SSEs are converted into alloys or slags, making it difficult to achieve effective separation of cathode materials and SSEs.^[^ ^220^, ^221^ ^]^ Therefore, the pyrometallurgical process is not an optimal recycling technology for ASSLMBs, as it not only consumes significant energy and has a complicated separation and purification process but also causes damage to the structure of the cathode and SSEs. In addition, this recycling technology is more suitable for the recycling of LIBs with high cobalt content in portable electronic devices but is less attractive for use in low‐cobalt‐content and cobalt‐free Li‐based batteries. n

### 4.3 Hydrometallurgical Process

Compared with pyrometallurgical process, hydrometallurgical process has significant advantages such as low energy consumption, high product purity, low CO_2_ emissions, and the ability to maximize the recovery of valuable metals in LIBs.^[^ ^8^ ^]^ For the unreacted Li metal in ASSLMBs, pretreatment must be performed before wet recovery to avoid the violent reaction of Li metal in the leaching solution. In addition, for sulfur‐based SSEs, H_2_S toxic gas will be released due to the presence of water; for polymer‐based SSEs, a small amount of polymer can significantly change the viscosity of the leaching solution and affect metal precipitation, and it is impractical to separate polymers and lithium salts from the leaching solution; for oxide‐based SSEs, separation, and purification are difficult due to the similar chemical properties of the SSEs and cathode materials.^[^ ^220^, ^227^ ^]^ In summary, the hydrometallurgical process is not suitable for certain ASSLMB systems (e.g., sulfur‐ and polymer‐based SSEs) but may be possible for oxide‐based SSEs, while the complexity of SSEs and electrode mixtures limits its development.

### 4.4 Direct Regeneration

Direct regeneration is a nondestructive recycling technology that has developed rapidly and recently due to its outstanding advantages, such as a simple process, low cost, and environmental friendliness, and the feasibility of direct regeneration of various cathode materials has been proven at the laboratory scale.^[^ ^186^ ^]^ In general, SSEs do not experience structural destruction except for fractional decomposition during SEI formation; thus, the majority of SSEs can be recycled by direct regeneration. Previous works have demonstrated that the degradation of cathode materials mainly occurs on the surface or subsurface of crystalline structures, forming localized spinel phases and rock salt phases, making it possible to achieve direct regeneration of cathode materials by converting these distorted regions on the surface back to layered structures.^[^ ^184^ ^]^ According to the LIBs direct regeneration method and the characteristics of SSEs, it is reasonable to expect breakthroughs in the direct recycling of ASSLMBs in the near future.^[^ ^63^, ^187^, ^188^ ^]^ The direct regeneration methods of ASSLMBs mainly include dissolution‐precipitation and hydrothermal regeneration.

### 4.5 Direct Regeneration (Dissolution‐Precipitation)

Liquid‐phase synthesis has been confirmed to be an effective method for the synthesis of sulfur‐based SSEs, where precursor powders are dispersed in organic solvents (e.g., methanol, ethanol, acetonitrile, and tetrahydrofuran), followed by drying and heat treatment to obtain sulfur‐based SSEs powders.^[^ ^228^, ^229^ ^]^ Therefore, dissolution‐precipitation can be utilized as an effective direct regeneration method for sulfur‐based ASSLMBs without chemical degradation of the cathode material or SSEs in inexpensive and safe organic solvents such as ethanol or acetonitrile. Due to the small grain size and poor crystallinity of sulfur‐based SSEs after dissolution‐precipitation, the conductivity decreases by one or two orders of magnitude.^[^ ^226^, ^230^, ^231^ ^]^ Therefore, it is necessary to combine dissolution‐precipitation with heat treatment to regain the crystal phase and ionic conductivity of the original materials. In addition, spent cathode materials can be recovered by the direct regeneration methods mentioned in the previous chapter, such as solid‐state calcination and hydrothermal and molten‐salt methods.^[^ ^63^, ^187^, ^188^ ^]^ For Li‐metal anodes, the lithium will be completely depleted, or only a small amount of reactive lithium will remain at the end of life of ASSLMBs, so the recovery of ASSLMBs mainly focuses on the recovery of the cathode materials and SSEs.^[^ ^220^, ^221^ ^]^ Recently, Chen et al. regenerated Li_6_PS_5_Cl SSEs through dissolution‐precipitation in ethanol and heat treatment, where the cathode electrolyte interphase was removed with water, and then the cathode materials were regenerated by a hydrothermal method.^[^ ^226^ ^]^ In addition, certain polymer‐based SSEs, such as polyethylene oxide (PEO)‐based SSEs, can be directly regenerated by the dissolution‐precipitation method. In PEO‐based SSEs, PEO and LiTFSI are soluble in water, can be effectively separated from other battery components and electrode materials, and can be directly recovered by removing water by heating. However, for oxide‐based SSEs, due to the similarity of transition metal oxides in SSEs and cathode materials, direct regeneration by dissolution‐precipitation is challenging.

### 4.6 Hydrothermal Regeneration

Certain SSEs, such as Li_1+_ *_x_* Al*_x_* Ti_2−_ *_x_* (PO_4_)_3_ (LATP), have been successfully synthesized by hydrothermal regeneration methods.^[^ ^232^, ^233^ ^]^ In addition, hydrothermal regeneration has been demonstrated to be effective in the direct regeneration of cathode materials.^[^ ^184^, ^185^, ^195^ ^]^ Therefore, direct regeneration of ASSLMBs is possible based on the chemical similarity of oxide electrolytes and cathode materials. Furthermore, direct regeneration can be achieved without separation of interpenetrating or co‐sintered SSEs and cathode materials, and subsequent heat treatment helps to reestablish tight contact. In addition, Huang et al. recently proposed a green, sustainable deformation‐driven re‐sintering technology for spent garnet‐type SSEs, and the regenerated SSEs exhibited good electrochemical performance.^[^ ^234^ ^]^

It is important to incorporate battery recycling into the design of next‐generation Li‐based batteries, including intelligence‐assisted predesign strategies, sustainable electrodes, and electrode materials separation predesign strategies.^[^ ^235^, ^236^ ^]^ Specifically, intelligence‐assisted predesign strategies of next‐generation Li‐based batteries can provide battery health diagnosis data, identify battery assembly and composition, and establish the cost‐effective sorting and disassembly process.^[^ ^235^ ^]^ Optimization of battery intrinsic recyclability and sustainability is a prerequisite for battery recycling, such as the Co‐free electrodes and organic electrodes.^[^ ^235^ ^]^ Moreover, redesign for electrode material separation to achieve non‐destructive battery disassembly, access to pure electrode active materials is another important issue to be addressed in the direct regeneration process.^[^ ^235^, ^236^ ^]^ Although there are significant challenges to incorporate recycling into the design of next‐generation Li‐based batteries, the strong global demand for high energy density batteries and sustainability will force substantial improvements in next‐generation battery manufacturing before their commercialization.

The development trend of Li‐based batteries is a transition from liquid‐ to SSEs and from Li‐ion to Li‐metal chemistry. However, sustainability research on ASSLMBs is still in the early stages. To adapt to the current trend, it is necessary to design a recycling method or at least a guideline that is suitable for ASSLMBs according to the existing LIBs recycling methods and the characteristics of Li metals and SSEs. Therefore, the development of dedicated ASSLMBs recycling methods still requires considerable effort.

## 5 Conclusion and Perspective

It is well accepted that spent LIBs recycling can alleviate most of the existing concerns, providing economic, environmental, sustainable, and geographical benefits. Therefore, the recycling of spent batteries is critical. However, industrial‐scale recycling is hindered by two aspects: technical challenges and economic viability.

### 5.1 Technical Challenges

Currently, many breakthroughs have been made in LIBs recycling; however, the existing recycling technologies are still not ideal. The remaining challenges and limitations in the field of LIBs and next‐generation Li‐based battery recycling need to be solved. In addition, LIBs recycling technologies need to keep up with the development of battery technology to establish a flexible, economically feasible, and high‐recovery‐rate recycling technology. Listed below are the needs and challenges faced in different recycling technologies.

1) Disassembly and pretreatment: Differences in shape (e.g., cylindrical, prismatic, and pouch‐like), size (e.g., 18 650 and 26 650 for cylindrical batteries), and composition (e.g., LCO, NCM, and LFP) of LIBs present special challenges for disassembly and pretreatment. Safe and efficient disassembly and pretreatment are the top priority for avoiding potential hazards in future LIBs recycling. Therefore, proper labeling of LIBs chemistry by battery manufacturers facilitates efficient recycling of batteries according to single chemical composition. In response to this need, the Society of Automotive Engineers has developed a corresponding labeling standard (J2936) for LIBs. In addition, to make the battery dismantling process much easier and more efficient, the battery design should be modified for easy disassembly. That is, in the battery structure design and battery manufacturing process, we need to take battery recycling and battery dismantling processes into consideration to design a structure for easy disassembly in the future for reuse or recycling.
2) Pyrometallurgical process: Currently, pyrometallurgical processes for recycling LIBs have been commercialized, and the business model mainly includes portable electronic products with high‐Co‐concentration LIBs. However, this model will become more challenging for electric vehicle LIBs because they contain a low or zero cobalt content. Furthermore, in the pyrometallurgical process, both anode and organic materials are burned and cannot be recovered, and large amounts of toxic gases are produced. In addition, metals such as Al, Li, and Mn remain in the slag, and with the gradual increase in lithium price, it is becoming more urgent to develop technology to recover lithium from slag. Therefore, innovation is needed to adapt this process to cobalt‐free or low‐cobalt LIBs chemistries and to achieve efficient recovery of lithium and other battery components, such as developing new roasting conditions that make it easier to separate and purify valuable metals or other battery components.
3) Hydrometallurgical process: Hydrometallurgical processes are another currently commercialized technology and are used to recover cathode materials, while the economic value of graphite anodes and electrolytes is ignored. In addition, acid/alkali leaching is convenient and efficient but causes secondary pollution, increasing the overall cost, and bioleaching is environmentally friendly but cannot meet the actual demand. Therefore, it is necessary to develop novel green solvents as a substitute for traditional acid/alkali leaching solutions and new special technology‐assisted leaching methods to achieve mild leaching of metal ions and other LIBs components.
4) Direct regeneration: Direct regeneration is simpler to operate than pyrometallurgical and hydrometallurgical processes and does not require the destruction of LIBs cathode materials. Physical separation and posttreatment (i.e., relithiation and heat treatment) can repair the composition and structural defects of cathode particles, and then cathode material regeneration can be achieved. However, relithiation of degraded cathode materials usually requires operation at high temperatures or pressures, which brings challenges to the industrialization of direct regeneration. The influence of impurities on cathode materials is another important challenge for direct regeneration. In addition, direct regeneration is mainly designed for the cathode materials but ignores other valuable battery components, such as anodes and electrolytes. In the future, a mild process is needed to realize the synchronous regeneration of anodes, cathodes, and other components, forming a recycling loop inside a single battery.
5) Recycling of next‐generation Li‐based batteries: The strong adhesion and high reactivity of Li‐metal leads to difficult disassembly of Li‐metal batteries. In addition, the complete mixing between SSEs and electrodes (except Li‐metal anodes) makes it difficult to separate these components effectively. Conventional recycling technologies are not suitable for ASSLMBs as they not only destroy the crystal structures of the cathode and SSEs but also lead to complicated separation and purification processes due to the complex components. Direct regeneration technology has received extensive attention as a nondestructive recycling technology; however, exploration of next‐generation batteries is still in its infancy. Therefore, it is necessary to design a recycling method suitable for ASSLMBs.

### 5.2 Economic Viability

LIBs recycling technologies are in dynamic development, so recycling technologies need to keep up with the development of recycling; only in this way can the optimization of the recycling process and regenerated materials be guided to achieve maximum economic benefits. In addition, strengthening the cooperation between academia and industry and transforming the scientific research achievements of academia into industrial recycling technologies will help to improve the economic benefits of recycling.

1) Scaling up: Although researchers have proposed a variety of strategies to replace the traditional recycling process, based on the complexity and huge industrial scale, realizing the transition from academic research to industrial application still faces great challenges. The information mismatch between academia and industry will also limit the development of LIBs recycling technology. In addition, collection, storage, and transportation are critical to the industrialization of spent LIBs recycling. Therefore, in the face of the upcoming large amount of spent LIBs, it is of great significance to develop an economical and feasible new recycling technology to realize the industrial‐scale recycling of spent LIBs.
2) Economic benefits: The economic benefits of the recycling process are the basis for the survival of recycling enterprises. However, current recovery methods mainly focus on the recovery of cobalt in cathode materials, and the future development trend of the battery industry is based on the use of cathodes with less cobalt or even cobalt‐free cathodes, which poses challenges to the economic feasibility of traditional LIBs recycling. If the value of battery materials is sharply reduced, the economic benefits of battery recycling will also be reduced. Therefore, to ensure enterprise profits, the optimization or innovation of existing recycling techniques is necessary, such as simplifying the recycling process by recycling cathodes, reducing recycling costs, and reducing secondary pollution, among others. However, these considerations are only from an economic perspective; LIBs recycling will still play a major role due to benefits in other aspects.

As LIBs recycling is critical to maintain future supply chains and prevent major environmental pollution, it is necessary to develop cost‐effective, sustainable, and environmentally friendly recycling technologies. Although innovative LIBs recycling technologies (i.e., direct regeneration) have prominent advantages over conventional LIBs recycling technologies (i.e., pyrometallurgical and hydrometallurgical processes), they are still at the laboratory scale, and more research is needed to validate the results of small‐scale experiments. Furthermore, as LIBs are developing, it is necessary to understand the development trend of LIBs concerning recycling, which has important guiding significance for material recovery and recycling processes. Considering the growing collaboration between academia and industry in developing novel LIBs recycling technologies, it is reasonable to expect that a major breakthrough in sustainable LIBs recycling technology will be achieved in the near future.

## Conflict of Interest

The authors declare no conflict of interest.
